# Copahue Geothermal System: A Volcanic Environment with Rich Extreme Prokaryotic Biodiversity

**DOI:** 10.3390/microorganisms3030344

**Published:** 2015-07-08

**Authors:** María Sofía Urbieta, Graciana Willis Porati, Ana Belén Segretín, Elena González-Toril, María Alejandra Giaveno, Edgardo Rubén Donati

**Affiliations:** 1 CINDEFI (CCT La Plata-CONICET, Facultad de Ciencias Exactas—UNLP), calle 50 entre 115 y 116 N° 227 La Plata, Buenos Aires B8508, Argentina; E-Mails: willis.graciana@biotec.quimica.unlp.edu.ar (G.W.P.); ana.belen.segretin@gmail.com (A.B.S.); donati@quimica.unlp.edu.ar (E.R.D.); 2 Centro de Astrobiología, Instituto Nacional de Técnica Aeroespacial (INTA-CSIC), Carretera de Ajalvir Km. 4, 28850, Torrejón de Ardoz, Madrid 28850, Spain; E-Mail: gonzalezte@cab.inta-csic.es; 3 Laboratorio de Biolixiviación, Departamentoo de Química—Facultad de Ingeniería, Universidad Nacional del Comahue, PROBIEN (CONICET-UNCo) Buenos Aires 1400 (8300) Neuquén, Argentina; E-Mail: agiaveno@hotmail.com

**Keywords:** Copahue geothermal system, acidic environment, prokaryotic biodiversity, extremophiles

## Abstract

The Copahue geothermal system is a natural extreme environment located at the northern end of the Cordillera de los Andes in Neuquén province in Argentina. The geochemistry and consequently the biodiversity of the area are dominated by the activity of the Copahue volcano. The main characteristic of Copahue is the extreme acidity of its aquatic environments; ponds and hot springs of moderate and high temperature as well as Río Agrio. In spite of being an apparently hostile location, the prokaryotic biodiversity detected by molecular ecology techniques as well as cultivation shows a rich and diverse environment dominated by acidophilic, sulphur oxidising bacteria or archaea, depending on the conditions of the particular niche studied. In microbial biofilms, found in the borders of the ponds where thermal activity is less intense, the species found are completely different, with a high presence of cyanobacteria and other photosynthetic species. Our results, collected during more than 10 years of work in Copahue, have enabled us to outline geomicrobiological models for the different environments found in the ponds and Río Agrio. Besides, Copahue seems to be the habitat of novel, not yet characterised autochthonous species, especially in the domain *Archaea*.

## 1. Introduction

Prokaryotic biodiversity in geothermal environments has been studied for many years, since the height of research in Yellowstone National Park in the early 70s [1,2]. In all that time, the focus of interest has expanded from mere curiosity of knowing the species that thrived in environments with extreme conditions [3] to understanding their community structure and ecological role [4,5], to finding their connection with the origin of life on Earth or even with other planets [6,7], to searching novel extremophilic microorganisms that could be used to develop or improve biotechnological processes [8,9,10,11]. With so much potential at hand, the need for studying new areas became clear and the research of diverse geothermal environments around the world increased greatly. The case of acidic geothermal fields was particularly specific, as they are usually associated with active volcanoes where the geochemistry is mainly related to sulphur and iron minerals. The biodiversity of those environments is especially interesting due to the presence of acidophilic species that are able to obtain energy from the oxidation or reduction of such minerals, in many cases autotrophically. Iceland and Kamchatka are examples of this kind of environments, where much research on biodiversity has been done ([12,13,14,15] as a few examples); however, there are not many other reports on the subject.

The Copahue geothermal system constitutes an extreme environment with a variety of acidity and temperature conditions that is dominated by the still active Copahue volcano. It presents diverse geothermal manifestations such as mud cones, hot springs, ponds, and pools; furthermore, a very acidic river rises close to Copahue’s crater and flows slowly downstream. The aim of this article is to build, based on our previous results, a comprehensive review of the microbial biodiversity of the Copahue geothermal system.

## 2. Description of the Copahue Geothermal System

**Figure 1:**
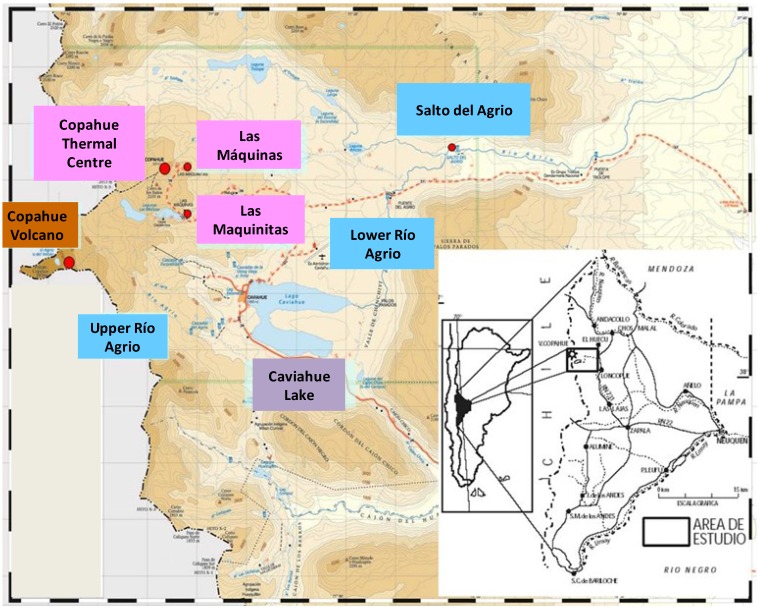
Schematic map of the most representative points in Copahue geothermal system, including the locations described in this work.

The Copahue-Caviahue geothermal system is located in the northwest corner of Neuquén province in Argentina, in the northern Patagonian Cordillera bordering Chile. Figure 1 shows the locations reported in this work as well as the geographic location of the Copahue geothermal system in Argentina.

**Figure 2:**
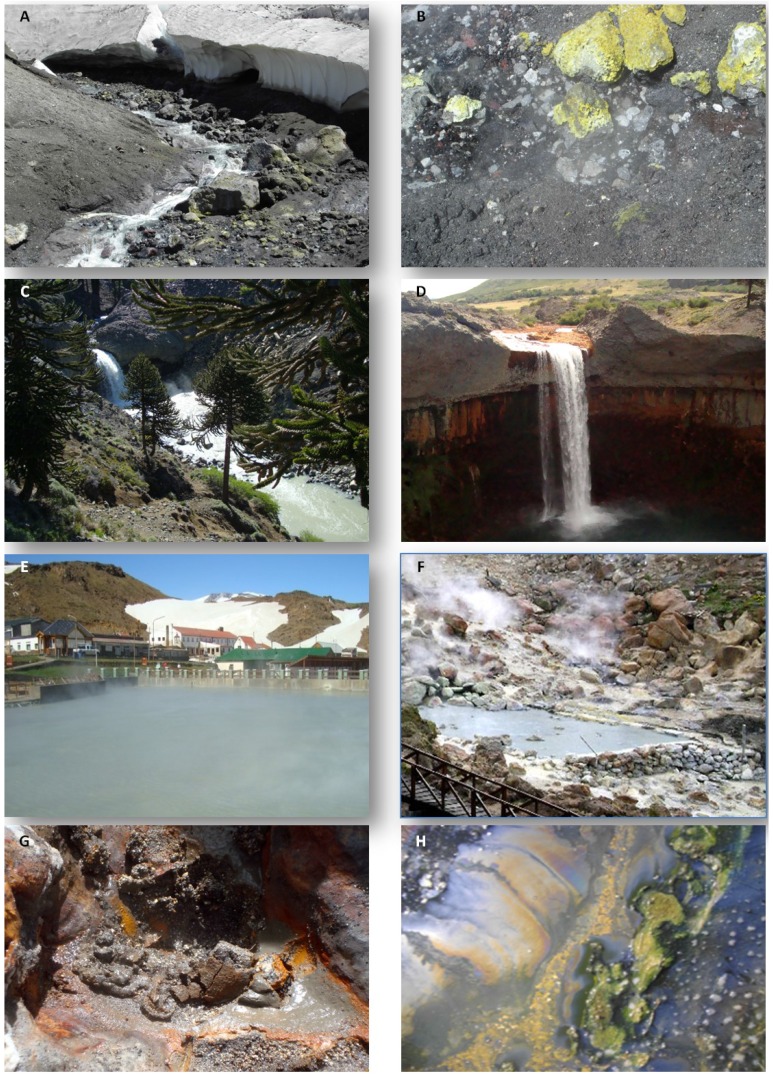
(**A**) Upper Río Agrio (URA) near its geothermal sources; (**B**) Sulphur deposits in the margins of URA; (**C**) URA in the area of the waterfalls; (**D**) Waterfall ‘Salto del Agrio’ in Lower Río Agrio where the precipitates of ferric iron can be noticed; (**E**) Copahue Thermal Centre; (**F**) Las Maquinitas; (**G**) Anaerobic sediments; (**H**) Microbial biofilms.

The Copahue geothermal system owes its particular characteristics to the still active Copahue volcano located at 2799 meters above sea level (m.a.s.l). Its acidity and varying temperature conditions make it especially interesting for conducting biodiversity studies and for exploring novel extreme microorganisms. Copahue is located in a depressed area of 300 km^2^ where the movement of tectonic grabens has given rise to diverse geothermal manifestations such as mud cones, hot springs, ponds, and pools that are grouped into five main areas named Copahue Thermal Centre, Las Máquinas, Las Maquinitas, Anfiteatro, and Chancho-Co (over the Chilean side). Geological studies have determined that the waters of the geothermal reservoir are acidic and include high concentrations of ions coupled with an abundant presence of sulphur [16]. In the following sections, we describe the main geochemical characteristics of the three acidic thermal manifestations where our research group has performed microbial biodiversity studies over the past 10 years: Las Máquinas, Las Maquinitas, and the Copahue Thermal Centre (Figure 1 in pink). The specific physicochemical characteristics of the sampling points are listed in Table 1; images of the area are shown in Figure 2 (E to H). Las Máquinas is the most pristine site in the Copahue geothermal field and includes various ponds and hot springs as well as a large pool with acidic waters and moderate temperatures with sulphur and pyrite deposits on its walls [17]. Las Maquinitas is the smallest of the thermal manifestations but the most extreme one, with ponds and hot springs with temperatures near water boiling point at Copahue’s elevation (approx. 90 °C) and pH values of around 2 and with visible traces of crystallised sulphur on the rock walls which are the product of volcanic gases escaping from the vents [18]. The Copahue Thermal Centre is the largest of the manifestations with ponds, vents, and lagoons with different temperatures and pH conditions that create different environments, some of them with extensive development of algae and biofilms. A healthcare facility operates in part of this area using artificial pools containing mud and water drained from the naturally acidic geothermal ponds.

**Table 1:** Most relevant physicochemical parameters of the water of the ponds, the microbial biofilms, and waters of Río Agrio analysed. A brief explanation of the abbreviated names of the points at Río Agrio is given at the bottom of the table. ND: not detected; -: not measured.

Sampling Point	Abbreviation	T (°C) ^a^	pH ^a^	Conductivity (μS cm^−1^) ^a^	SO_4_^2−^ (mg/L) ^b^	Cl^−^ (mg/L) ^c^	Fe (mg/L) ^d^
**Water samples**							
Las Máquinas	LMa	36.0	3.2	663	119.5	5.30	7.0
Laguna Verde Este	LVE	31.5	3.0	1317		35.7	3.3
Baño 9	B9	40.5	2.7	3720		2.10	7.0
Laguna Sulforosa	LS	54.3	3.0	1133	291.8	56.7	3.7
Las Maquinitas	LMi	87.0	2.0	4520	346.8	1.80	32.8
**Biofilm samples**							
Las Máquinas	LMa biof	36.0	4.8	-	ND	ND	ND
Laguna Verde Este	LVE biof	30.0	4.8	-	ND	ND-	ND
Baño 9	B9 biof	30.0	2.7	-	ND	ND	2.4
Las Maquinitas	LMi biof	35.0	3.5	-	ND	ND	0.4
**Rio Agrio**							
Vertiente Río Agrio	VA2 ^1^	29.0	1.0	15,450	6226.1	6780.2	560
Unión dos Vertientes	U2V ^2^	9.0	1.5	10,690	2467.1	3567.3	470
Río Agrio Superior	AS3 ^3^	6.7	1.7	11,690	3274.3	4.7	111
Cavellera de la Virgen	CV ^4^	15.9	2.0	3290	875.4	586.7	30
Caviahue Lake	CL ^5^	8.3	2.0	946	266.0	48.2	7
Salto del Agrio	SA ^6^	16.9	3.6	516	1481.6	30.4	3

^a^: Temperature, pH and conductivity were measured *in situ* with a Hanna HI 8424 NEW portable instrument accurately calibrated against calibration standards; ^b^: The concentration of sulphate was determined by a turbidimetric method using an excess of barium chloride; ^c^: The concentration of chloride was determined by titration with mercuric nitrate solution in the presence of diphenylcarbazone bromophenol blue indicator; ^d^: Soluble Fe determined on filtered water samples by atomic absorption spectrophotometry using a Shimadzu AA-6650 spectrophotometer; ^1^: VA2: one of the two acidic thermal springs that are considered the origin of Río Agrio; ^2^: U2V: the point where the two original streams meet and form Upper Río Agrio (URA); ^3^: AS3: a point in the middle part of URA in the area of the snowmelt tributary streams; ^4^: CV: a point in the last part of URA in the area of the waterfalls before it discharges in Caviahue Lake; ^5^: Caviahue Lake; ^6^: SA: the big waterfall found in Lower Río Agrio.

Another very interesting extreme environment found in this geothermal zone originates in the proximity of the crater of the Copahue volcano; approximately 100 m below there are two acidic thermal springs, with pH values between 0.3 and 2.3 which are considered the sources of acidic Río Agrio (they are referred to as VA1 and VA2 from the Spanish words “Vertiente del Agrio”). The temperature at the source of Río Agrio varies over time depending on the degree of volcanic activity, ranging approximately from 70 °C during periods of high activity to close to 20 °C, when the activity is less intense. On the other hand, the pH value of the waters is always very low, oscillating between 0.5 and 1 [19]. The streams from VA1 and VA2 converge a few meters below and form the Upper Rio Agrio (URA) that flows downslope from the Copahue volcano forming various waterfalls and ultimately discharging to the northern arm of Caviahue Lake 13.5 km downstream (Figure 1). Once the river flows away from its geothermal sources, the temperature of the water decreases greatly due to the joining of tributaries with meltwater; however, the waters still remain very acidic, with pH values close to 1. Other interesting physicochemical parameters of the first part of URA are the high conductivity and high concentrations of sulphate, chloride, and iron (see Table 1). Some kilometers downstream, URA changes its flow and geothermal composition due to the three main tributary streams that causes dilution and a gradual increase in pH up to 2. In our biodiversity studies, we have analysed various points of the middle part of URA with temperatures between 7 and 10 °C and pH values between 1.7 and 2 (Table 1 shows the physicochemical data of a sampling point identified as AS3). Lastly, the URA discharges to the Caviahue Lake, located at 1600 m.a.s.l. where the touristic village Caviahue is located on its west margin. The lake is from glacial origin and the waters are acidic with pH values between 2.1 and 3.7 and temperature of approx. 8 °C [20]. Due to the input from other tributary streams, mainly from snowmelt, the lake suffers a marked dilution of the elements that are present in the URA [20]. Lower Río Agrio (LRA) is the only effluent of Caviahue Lake. It continues its course to the north and leads to a spectacular waterfall named “Salto del Agrio” 5 km away (Figure 2D). At this point, the LRA widens and the margins become stained in an orange-reddish colour due to the precipitation of ferric minerals caused by the increase in pH (see sampling point SA in Table 1). The LRA continues to increase its pH gradually until it reaches neutrality 40 km downstream.

Our research group has worked on the microbiology of Copahue, focusing on prokaryotic biodiversity assessments through culture-independent approaches using microbial ecology techniques such as amplification and sequencing of the complete 16S rRNA gene of the entire community and FISH (Fluorescence *In Situ* Hybridisation) or its more sensible version CARD FISH (Catalysed Reported Deposition Fluorescence *In Situ* Hybridisation) for quantitative information on the species distribution and community structure [21,22,23]. Additionally, we tried to isolate and characterise autochthonous extremophilic microorganisms [24,25,26]. The ultimate goal of our research work sought to find novel species that would help develop new biotechnological procedures, or improve existing ones, mainly related to biomining, bioremediation of heavy metal contaminated sites and acid mine drainage and the production of alternative biofuels.

## 3. Prokaryotic Biodiversity at Copahue Geothermal Field

We have proposed the study of prokaryotic biodiversity in the Copahue geothermal field through two complementary strategies. On the one hand, we performed comprehensive non-culture based studies on the whole prokaryotic community of different ponds that were selected to represent the diverse temperature and pH characteristics found in the area (Table 1) as well as the influence of anthropogenic intervention [22]. The assessments of water samples as well as the microbial biofilms that develop at the edges of the rocks where thermal activity is less intense were conducted by amplification and sequencing of the partial 16S rRNA gene of bacteria and archaea (the set of primers used was 8F: 5′-AGAGTTTGATC(A/C)TGGC-3′ for *Bacteria* and 25F: 5′-TCYGGTTGATCCYGCCRG-3′ for *Archaea*; reverse primer for both was 1492r: 5′-TACCTTGTTACGACTT-3′ [27,28]). The fluorescence techniques FISH or CARD FISH were used when the quality of the samples allowed so. Hybridisations were conducted with general bacteria and archaea probes or more specific probes according to the phylogenetic classes or genera detected (the probes used are listed in Table 2). On the other hand, we conducted diverse enrichment cultures and isolations from samples with specific environmental characteristics pointing to the isolation of microorganisms with certain metabolic features, such as iron and/or sulphur compound oxidation, sulphate reduction, heavy metal transformation and photosynthesis, in connection with lipid accumulation or the production of other alternative sources of energy.

**Table 2:** Fluorescence labelled oligonucleotide probes used for FISH and CARD-FISH. Abv: abbreviations used in the text and figures.

Probe	Abv	Target	Target Sequence (5′-3′)	(%) FM ^a^	Specificity	Reference
EUB338I	EUB	16S	GCTGCCTCCCGTAGGAGT	0–35	*Bacteria* domain	[29]
EUB338 II		16S	GCAGCCACCCGTAGGTGT	0–35	*Planctomyces*	[30]
EUB338III		16S	GCTGCCACCCGTAGGTGT	0–35	*Verrumicrobia* (and others)	[30]
ALF968	ALF	16S	GGTAAGGTTCTGCGCGTT	20	*Alphaproteobacteria*	[31]
BET42a ^b^	BET	23S	GCCTTCCCACTTCGTTT	35	*Betaproteobacteria*	[32]
GAM42a ^c^	GAM	23S	GCCTTCCCACATCGTTT	35	*Gammaproteobacteria*	[32]
NTR712 ^d,e^	NTR	16S	CGCCTTCGCCACCGGCCTTCC	35	*Nitrospirae* group	[33]
ACD840	ACD	16S	CGACACTGAAGTGCTAAGC	10	*Acidiphilium* genus	[34]
TM1G0138	TM	16S	GCAGTTATCCCCCATCAAT	40	*Thiomonas* group 1	[35]
TM2G0138		16S	GTAGTTATCCCCCATCACA	40	*Thiomonas* group 2	[35]
THIO1	THIO	16S	GCGCTTTCTGGGGTCTGC	35	*Acidithiobacillus* spp.	[36]
ARQ915	ARCH	16S	GTGCTCCCCCGCCAATTCCT	20	*Archaea* domain	[37]
NON338		-	ACTCCTACGGGAGGCAGC	35	Negative control	[29]

^a^: Formamide percentage (vol/vol) in the hybridisation buffer; ^b^: Used in conjunction with a competitor probe, GAM42a (5′-GCCTTCCCACATCGTTT-3′) [32]; ^c^: Used in conjunction with a competitor probe, BET42a (5′-GCCTTCCCACTT CGTTT-3′) [32]; ^d^: Used in conjunction with a competitor probe, NTR712c (5′-CGCCTTCGCCACCGGTGTTCC-3′) [33]; ^e^: The complete name of this probe in the work of Daims *et al.* [33] is S-*-tspa-0712-a-A-21.

**Figure 3:**
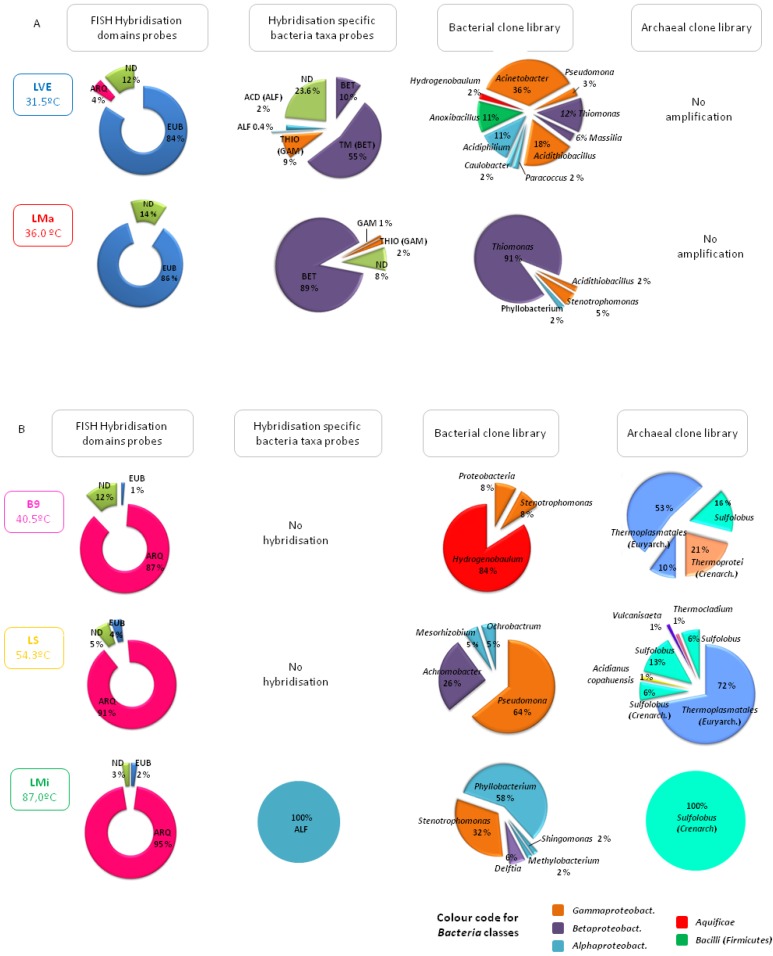
Representation of the biodiversity found by molecular ecology techniques in the waters of the Copahue geothermal ponds. (**A**) moderate temperature ponds; (**B**) higher temperature ponds.

Ponds and hot springs from the thermal manifestations hosted different community structures depending essentially on temperature but also on other factors such as anthropogenic intervention (Figure 3). A clear example is the different composition of the prokaryotic community found in two ponds, LVE and LMa, with similar acidic pH (approx. 3) and moderate temperature conditions (31.5 and 36 °C, respectively). As shown in Figure 3, both were clearly dominated by bacteria with over 84% hybridisation with the probes EUB I-III that together cover most of the species in the domain *Bacteria*. However, LMa, the pool located in the pristine area of Las Máquinas was colonised by acidophilic, mesophilic, autotrophic or mixotrophic sulphur oxidising bacteria related to the genera *Thiomonas* (91% of the clones analysed) and *Acidithiobacillus* (5%) while in LVE, the pool at Copahue Thermal Centre located inside the health care facility, the number of species detected was higher, with a smaller representation of acidophiles and the presence of various species of heterothrophs, such as *Acinetobacter* and *Pseudomonas*, which are related to human presence. As regards archaea, it was not possible to detect any in either of these moderate temperature ponds by amplification of 16S rRNA genes. Interestingly, as the temperature of the studied ponds increased, the prokaryotic community composition shifted towards archaea. For instance, in the waters of the pond Baño 9 (temperature of 40.5 °C and a pH of 2.7), also in the Copahue Thermal Centre manifestation but far away from the health care facility, archaea represented 84% of all the microorganisms detected according to DAPI staining and hybridisation with the probe ARQ915 specific for the domain *Archaea*. More than 60% of the archaeal clones were affiliated with the order *Thermoplasmatales* in the phylum *Euryarchaeota* (no further taxonomic classification was possible) and showed between 96% and 99% similarities with other uncultured archaeal clones found in other acidic geothermal regions around the world. The archaeal community was completed by two groups of creanarchaeotas: one classified only as members of the class *Thermoprotei* and the other associated with the genus *Sulfolobus* which are aerobic, thermophilic, acidophilic sulphur oxidising archaea very common in acidic high temperature environments [2]. The little bacteria detected in B9 were related to the genus *Hydrogenobaculum*, a group of acidophilic, thermophilic, chemolithotrophic, hydrogen and sulphur oxidising species found in many sulphur-rich geothermal environments [15,38] (see reference [22] for more details). In the acidic pond LS in the Copahue Thermal Centre with a temperature of 54.3 °C, *Thermoplasmatales* and *Sulfolobus* were still dominant and *Acidianus copahuensis* appeared. *A. copahuensis* is a novel thermophilic, acidophilic, facultative anaerobic, sulphur and iron oxidising strain that is apparently autochthonous to Copahue [26]. In pond LS, other thermophilic anaerobic archaea, such as *Vulcanisaeta* and *Thermocladium*, were detected. The most extreme conditions were found at Las Maquinitas, where one of the hot springs sampled had a temperature of 87 °C, close to the boiling point of water at Copahue’s altitude, and a pH of 2. Such extreme conditions were reflected in the biodiversity that was found: archaea represented 95% of all microorganisms detected by DAPI and hybridisation assays and were represented by only one sequence affiliated to the genus *Sulfolobus* that showed low sequence similarity (approx. 94%) to uncultured archaeal clones retrieved from diverse acidic and high temperature thermal environments, which might indicate the existence of another novel strain autochthonous to Copahue. On the other hand, bacteria detected in Las Maquinitas by amplification and sequencing of the 16S rRNA gene were all related to mesophilic and neutrophilic species typical of the soil or associated with human presence that were probably not metabolically active in the extreme environmental conditions of such hot springs.

Microbial biofilms tend to develop at the edges of pools and ponds where thermal activity is less intense. The prokaryotic biodiversity of these biofilms, which present temperature conditions around 30 to 36 °C (see Table 1), was completely different to what was found in the waters of the ponds. According to our 16S rRNA gene analysis, the microbial biofilms presented an important proportion of photosynthetic species and almost no presence of sulphur oxidisers [23]. The nature of the photosynthetic species is apparently determined by the pH of the microbial biofilm: in the samples with pH lower than 4, the only photosynthetic species detected were related to the eukaryotes *Bacillariophyta* and *Chlorophyta* while in the samples with higher pH values *Cyanobacteria* related to the genera *Synechococcus*, *Leptolyngbya*, *Mastigocladus* and *Fischerella*, and *Chloroflexi* of the genera *Roseiflexus* and *Chloroflexus* were also detected. As shown for archaeal species, many of the sequences related to photosynthetic species presented low similarities with known species, which opens the door to possible novel members of those genera. As regards the presence of sulphur oxidising bacteria in the microbial biofilms, only a small percentage of sequences related to *Thiomonas* were detected in the samples with more acidic conditions. The near absence of sulphur oxidisers could be correlated with the very low, or even undetected, levels of sulphate in those samples [23].

The species found by cultivation from different ponds from the Copahue geothermal field [25,26] correlate with the biodiversity detected in the molecular ecology assessments. For instance, sulphur oxidising species related to *Acidithiobacillus thiooxidans* and *At. caldus* were isolated from various moderate temperature ponds from the Copahue geothermal system while archaea related to *Sulfolobus* and *Acidianus* were isolated from more elevated temperature ponds. Heterotrophic acidophilic species, mesophiles and thermophiles, related to *Acidiphilium*, *Sulfobacillus*, *Alicyclobacillus*, and *Mesoaciditoga*, were isolated from all the samples tested [25]. A noticeable difference between both approaches to determining the prokaryotic biodiversity in Copahue is that while iron oxidisers were scarcely detected by molecular ecology techniques, species such as *At. ferrooxidans*, *Leptospirillum*, and *Sulfobacillus thermosulfidooxidans*, were isolated by cultivation from various samples when mineral growth medium supplemented with ferrous iron was used.

Bacteria with sulphate reducing activity were found during cultivation using anaerobic sediments on specific growth media. [39]. At present, we are working on the physiological characterisation of two strains isolated on overlay plates with basal salt medium supplemented with glycerol at pH 3 and incubated at 30 °C under anaerobic conditions. One of the two species presents 96% similarity to the isolate *Peptococcaceae* bacterium CL4, an acidophilic sulphate reducing bacterium isolated from the bottom layer of an acidic metal-rich stream in an abandoned mine in the Iberian Pyrite Belt in Spain [40], and the other is related to the genus *Desulfotomaculum* which are anaerobic mesophilic or thermophilic spore forming, sulphate reducing bacteria typical for subsurface environments [41]. Their ability to grow at very low pH values—in comparison to most sulphate reducing microorganisms—would make them especially useful for the bioprecipitation of heavy metals in the bioremediation of contaminated waters.

Photosynthetic species were also detected when samples from microbial biofilms or mats that developed near the ponds were inoculated in basal salt medium specific for microalgae and cyanobacteria enrichment cultivated at 25 °C, with a light period of 12 h. Species related to the cyanobacteria *Synechococcus elongates* and *Leptolyngbya* sp. and the microalgaes *Scenedesmus obliquus* and *Chlorella* sp. from the phylum *Chlorophyt*a were identified.

**Figure 4:**
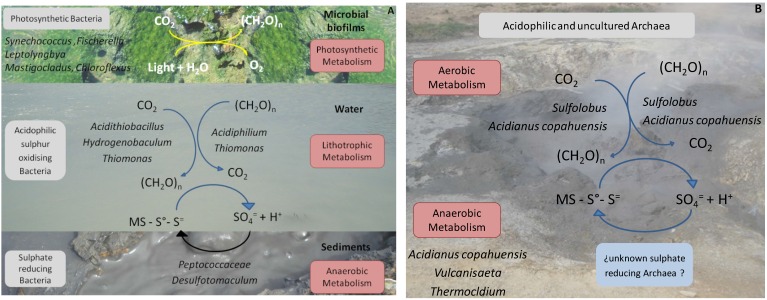
Schematic representation of the geomicrobiological models proposed for moderate (**A**) and high (**B**) temperature ponds in Copahue based on the species found. In (**A**) three different niches are represented: microbial biofilm, water, and sediments of the ponds. (CH_2_O)n: organic compounds; MS: metal sulphide. Modified from [22].

The biodiversity findings obtained through the qualitative and quantitative molecular ecology tools mentioned in addition to the use of biostatistics software to correlate these with the most relevant physicochemical parameters of the environment, such as temperature, pH and ion sulphate concentration (the end product of biological sulphur compounds oxidation) as well as the known and reported metabolic capacities of the microorganisms detected, allowed us to propose a biogeochemical model for the moderate and the high temperature ponds, which is schematised in Figure 4A,B, respectively.

According to our results, the environment of the moderate temperature ponds presents three different biological niches dominated by different bacteria with different preponderant metabolisms. Water in the ponds seems to be dominated by a lithoautotrophic type of metabolism supported by sulphur oxidation, both autotrophically by species such as *Acidithiobacillus* and *Hydrogenobaculum* that might act as the primary producers of the waters and mixotrophically by *Thiomonas* and *Acidiphilium*. The high concentration of sulphate (the end product of sulphur compounds oxidation) measured in the waters emphasises the importance of sulphur metabolism. In the sediments of the ponds which are under anaerobic conditions, sulphate reducing bacteria, such as members of the family *Peptococcaceae* and the genus *Desulfotomaculum*, use the sulphate produced by sulphur oxidiser and close the sulphur cycle. The bulk of the primary production seems to occur within the microbial films, mostly by photosynthetic bacteria such as *Synechococcus*, *Leptolyngbya*, *Mastigocladus* and *Fischerella* among *Cyanobacteri*a and *Roseiflexu*s and *Chloroflexus* among *Chloroflexi*. In the high temperature ponds, where no biofilms were found, the oxidation of sulphur compounds by autotrophic and/or mixotrophic thermoacidophilic archaea, such as *Sulfolobus* and *A. copahuensis*, seems to be the dominant type of metabolism. As in the moderate temperature waters, the aerobic oxidation of sulphur compounds by acidophilic bacteria and archaea releases protons that keep the low pH of ponds and hot springs. Regarding anaerobic metabolisms, some thermoacidophilic anaerobic archaea from the genera *Vulcanisaeta* and *Thermocladium* were found that are able to reduce sulphur and other sulphur compounds. In spite of trying different isolation strategies, there is still no record of archaea with sulphate reducing ability in Copahue. However, it is important to consider that many of the archaeal sequences retrieved from the high temperature ponds presented low sequence similarity with know and well-characterised species, indicating that their metabolic features are yet to be discovered [22].

When comparing the biodiversity in Copahue with that reported for other environments, certain similarities were found. For instance, Benson and co-workers [42] studied microbial species inhabiting sulphur, non sulphur and iron rich acid high temperature geothermal steam vents at different locations around the world; according to their results archaea from the domain *Crenarchaeota* were dominant in all the steam vents analysed and species from the genus *Sulfolobus* prevailed in sulphur or iron-rich spots, as found in Copahue. In the acidic geothermal field of Ohwakudani in Hanoke, Japan, Kato and co-workers [43] reported similar archaeal species to the ones found in Copahue; however, the abundances and distributions were different. In the highest temperature hot springs, *Crenarchaeota* were also dominant, but the species found were related to the genera *Vulcanisaeta* and *Caldivirga* with a minor contribution of *Sulfolobus*. Similar to Copahue, *Thermoplasmata* (*Euryarchaeota*) were reported in the less extreme temperature ponds; however, in the moderate temperature ponds of the Japanese geothermal field, thermophilic archaea related to *Metallosphaera* and *Acidianus* were found, whereas in moderate temperature ponds in Copahue, no archaea were detected. Another notorious difference between the archaeal clone libraries from both geothermal environments is that in the Japanese study, the archaeal sequences presented high similarity to cultivated species while in Copahue, most of the archaeal clones were distantly related to cultivated species, and in some cases even to uncultivated ones, which points to the existence of novel autochthonous species. The Kawah Hujan B geothermal field in Indonesia presents physicochemical conditions similar to those in Copahue (pH values of 2, high temperature, and presence of sulphur compounds and high concentration of sulphate ion) and the species found there were affiliated to heterotrophic *Proteobacteria*, *Firmicutes*, especially *Alicyclobacillus* and *Crenarchaeota* [44].

## 4. Prokaryotic Biodiversity at Río Agrio

**Figure 5:**
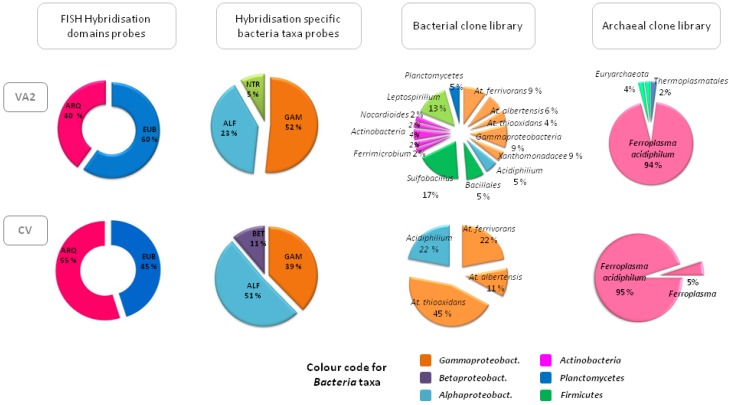
Representation of the biodiversity found by molecular ecology techniques in the source or Upper Río Agrio (VA2) and near its discharge in Caviahue Lake (CV).

In the case of the acidic Río Agrio, the approach used to assess its biodiversity involved culture and non-culture based techniques in addition to sequencing of the almost complete 16S rRNA gene of *Bacteria* and *Archaea* with the same primers described in Section 3 and CARD-FISH hybridisation with some of the probes listed in Table 2. Along URA we detected approximately the same number of microorganisms: around 2.5 × 10^6^ cells/mL according to DAPI staining counts. Figure 5 summarises the biodiversity found at the source of the river (point VA2) and in one of the waterfalls next to the discharge of the URA in Caviahue Lake (point CV). The figure shows how the ratio of *Bacteria/Archaea* remains approximately constant through the URA as well as the distribution of bacterial classes, with a clear dominance of *Gamma*- and *Alphaproteobacteria*. *Gammaproteobacteria* were represented mostly by different *Acidithiobacillus* species such as *At. ferrivorans*, *At. albertensis*, and *At. thiooxidans*. In addition, we found a minor fraction of sequences that could not be classified beyond the class *Gammaproteobacteria*, although they showed 99% sequence similarity to other sequences retrieved from diverse natural or mining-related acidic environments. *Alphaproteobacteria* was only represented by one species belonging to the genus *Acidiphilium*, a group of acidophilic chemoorganotrophic bacteria able to oxidise sulphur compounds that are generally found in acidic environments where chemolithotrophic species are found [45]. *Leptospirillum* was the only species found within the *Nitrospira* group, but only in the first part of the URA where the concentration of soluble ferrous iron (its only energy source) was higher. At the source of the river where the pH equals 1, the *Leptospirillum* species would be metabolically favoured for the oxidation of ferrous iron over the *Acidithiobacillus* species, whose enzymatic routes become inhibited at pH values lower than 1.3 [45]. Still, *Acidithiobacillus* species seemed to be more abundant than *Leptospirillum* along the URA, probably because sulphur compounds (which can be used as a source of energy by the former but not by the latter) are much more abundant than ferrous iron. Close to the source of Río Agrio, 6%–8% of sequences related with *Sulfobacillus*, a sulphur oxidising species commonly found to be a member of the microbial community in acid mine drainage were also detected [46]. Also detected were some sequences that were related to *Ferrimicrobium*, an acidophilic heterotroph capable of oxidising ferrous iron and reducing ferric iron under anaerobic conditions [47]. Near the end of the URA in the area of the waterfalls where the pH is fixed at a value of 2, the number of species dropped, being *Acidithiobacillus, Acidiphilum* and *Ferroplasma* the only species detected (see Figure 5). As regards the archaeal population along the URA, over 94% of the clones analysed were related to *Ferroplasma*. *Ferroplasma* species are acidophilic, lithoautotrophic or mixotrophic *Euryarchaeota* that are capable of oxidising iron and pyrite and which are important members of the microbial community in very acidic, heavy metal-rich environments [48].

Our results show that the microbial community along Río Agrio consists of a relatively small number of acidophilic species that are commonly found in acid mine drainage [49] or in natural acidic environments such as Rio Tinto [36]. In addition, a lack of correlation in the canonical analysis of the most significant physicochemical parameters (temperature, pH, iron and sulphate concentrations listed in Table 1) and the collected biodiversity data was found; this result seems to indicate that the structure of the microbial community and the species found along its course does not change significantly in spite of the dilution that the river suffers due to the input of tributary streams from snowmelt [50].

The cultivation approach allowed isolating acidophilic mesophilic iron oxidising species related to *At. ferrooxidans* and *Leptospirillum* when samples from the area of the waterfalls of URA were inoculated in basal salts solutions supplemented with ferrous iron and cultivated at 30 °C [24]. In a comprehensive assessment of the biodiversity by cultivation under different conditions, microorganisms with diverse metabolic capabilities were detected in samples from different stations along the URA [25]. Mesophilic iron oxidising bacteria (*At. ferrooxidans* and *L. ferrooxidans*) were isolated from samples in the area of the waterfalls, while thermophilic iron oxidising archaea (*Sulfolobus* and *Acidianus*) were isolated from the samples collected near the source of the river. Sulphur oxidisers, such as *At. thiooxidans* and *At. caldus*, were isolated from samples all along the river. No acidophilic heterotrophic microorganisms were isolated from Rio Agrio samples, which further emphasises that the prevailing primary producers are lithoautotrophic species. [50].

**Figure 6:**
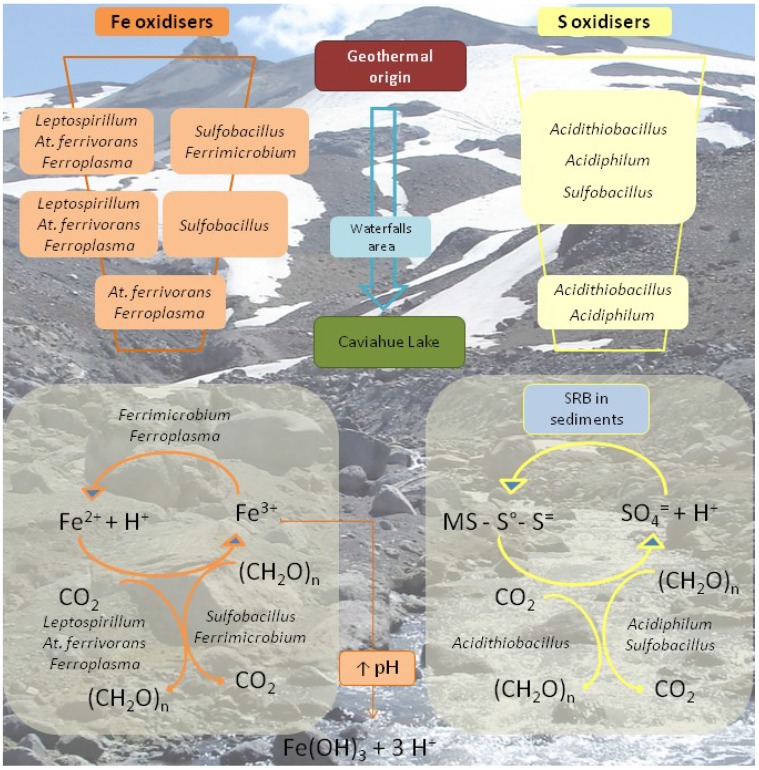
Schematic representation of the most important microbial metabolisms found in the Upper Río Agrio. The model intends to show the disappearance of certain species as the river goes down Copahue volcano slope and the physicochemical conditions change. SRB: sulphate reducing bacteria; (CH_2_O)n: organic compounds; MS: metal sulphide. Modified from [50].

As in the case of the Copahue geothermal field, Figure 6 outlines a geomicrobiological model of the upper part of Rio Agrio. The particular geology of the source of Rio Agrio and the sustained volcanic activity has led to the accumulation of ferrous iron compounds and even more of sulphur compounds. According to our results, the prokaryotic biodiversity found in the waters of URA seems to be determined by the species found in the origin of the river and that is why we found chiefly the same aerobic, mesophilic, acidophilic, chemolithoautotrophic sulphur and/or iron oxidising species along its course. Río Agrio is an environment with low organic carbon content and this is reflected by the almost absence of strict heterotrophic species. CO_2_-fixing chemilithoautotrophic species such as *Acidithiobacillus*, *Leptospirillum*, and *Ferroplasma* appear to be responsible for primary production. The presence of sulphur oxidising species, such as *Acidithiobacillus*, *Sulfobacillus*, and *Acidiphilium*, help to explain the maintenance of the acidic pH and the high amount of sulphate measured in all URA. Iron oxidising species (*Leptospirillum*, *At. ferrivorans*, *Ferrimicrobium*, *Sulfobacillus*, *Ferroplasma*) are much more diverse at the origin or URA where ferrous iron concentration is higher. *Leptospirillum*, the only strict iron oxidiser, disappeared quickly; in turn, only *At. ferrivorans* (also able to oxidise sulphur compounds) and *Ferroplasma* could be detected near the last stretch of the river where iron concentration is around 30 mg/L. The decrease in iron concentration can be explained by the precipitation of ferric iron hydroxy sulphate compounds (like jarosite) when the pH increases over 2. This process, which releases protons, acts as a buffer that also contributes to maintaining the low pH. Lastly, worth highlighting is the detection of many bacterial and archaeal sequences along the URA that could only be associated with the higher taxonomy levels and which showed 99% similarity to the sequences of uncultured clones that are found in diverse acidic environments. [50]. This might be related with the existence of a ubiquitous biodiversity of moderate temperature acidic environments yet uncharacterised.

In terms of biodiversity, Rio Agrio can be compared to Rio Tinto in the Iberian Pyrite Belt in Spain, another natural acidic river. The biodiversity of both acidic rivers is quite similar with the prevalence of acidic lithoautotrophic species, although Rio Tinto is dominated by iron oxidisers such as *Leptospirillum* and *At*. *ferrooxidans* due to the high concentration of soluble iron present (around 1000 mg/L) [51]. Archaeal species found in the river in Spain were also related to *Thermoplasmatales*, particularly *Ferroplasma*, but they were detected in smaller proportions than in Rio Agrio [36]. With the exception of these two examples, natural acidic rivers are not common, which means that further comparison needs to be made mostly with other kinds of aquatic environments of similar characteristics. Such is the case of acidic lake Kawan Ijen in Indonesia, where the 16S rRNA bacterial sequences reported were not related to the ones detected in Rio Agrio probably because of the very low pH (approx 0.5) at which neither *Acidithiobacillus* nor *Leptospirillum* can develop, in spite of the high iron concentrations present [52]. Acid mine drainages usually present microbial communities very similar to that found in Rio Agrio and represented mainly by *Acidithiobacillus*, *Leptospirillum*, *Sulfobacillus*, and *Ferroplasma* species [49,53,54].

## 5. Conclusions

This work is the first comprehensive review of the microbial biodiversity found at Copahue geothermal system which is a rich and diverse extreme environment with a wide range of temperatures and acidity. Different microbial metabolisms mainly related to sulphur cycle were detected in ponds and hot springs but also along the acid Río Agrio. Autotrophic bacteria and archaea were ubiquitous while heterotrophic microorganisms were mainly located in the ponds and hot springs where archaea seemed to dominate the high-temperature environments. Although many species detected or even isolated from this system are also present in similar environments, we found many 16S rRNA sequences, chiefly from the domain *Archaea*, distantly related to known and characterised species, which means that this system is a potential source of novel and possibly interesting species.
